# High-Sugar Consumption Induces Anxiety-Like Behavior via Activating the Glutamatergic Neurons in the Nucleus of the Solitary Tract in Mice

**DOI:** 10.3390/biology15080646

**Published:** 2026-04-19

**Authors:** Pingjie Wang, Yang Tan, Zhe Fan, Shilin He, Chunxiao Chen, Ying Sun, Wenghei Hong, Zihao Wang, Keke Zhang, Evandro Fei Fang, Yun Liu, Zili Zhang

**Affiliations:** 1 Department of Neurology, The First Clinical College of Zhengzhou University, Zhengzhou University, Zhengzhou 450052, China; 2 Department of Physiology, School of Medicine, Jinan University, Guangzhou 510632, Chinazzli2575@fsyyy.com (Z.Z.); 3 Department of Microbiology and Immunology, School of Medicine, Jinan University, Guangzhou 510632, China; 4 Department of Thyroid and Breast Surgery, Guangzhou Medical University Affiliated Women and Children’s Medical Center, Guangzhou 510623, China; 5 Key Laboratory of State Administration of Traditional Chinese Medicine, Department of Pathophysiology, School of Medicine, Jinan University, Guangzhou 510632, China; 6 Department of Clinical Molecular Biology, University of Oslo and Akershus University Hospital, 1478 Lørenskog, Norway; 7 The Norwegian Centre on Healthy Ageing (NO-Age) and the Norwegian National Anti-Alzheimer’s Disease (NO-AD) Networks, 0372 Oslo, Norway; 8 Reproductive Medicine Center, Foshan First People’s Medical Center (The Affiliated of Southern University of Science and Technology), Foshan 528000, China

**Keywords:** anxiety, chemogenetics, electrophysiology recording, glutamatergic neuron, sweetened beverages

## Abstract

Anxiety, a common mental disorder, is epidemiologically linked to high dietary sugar intake. However, the underlying neural mechanisms remain poorly understood. Here, using male C57BL/6 mice (*n* ≥ 10 per group), we show that two-week consumption of sugar-sweetened drinks reliably induced anxiety-like behavior, characterized by reduced center time in the open field test and less open arm exploration in the elevated plus maze. Notably, consumption of sucrose, glucose, or the non-metabolizable glucose analog methyl-α-D-glucopyranoside induced anxiety-like behavior, whereas intake of the artificial sweetener acesulfame potassium (Ace-K) did not. Moreover, after two weeks of exposure to sucrose or glucose but not to Ace-K, c-Fos expression was elevated in glutamatergic neurons of the nucleus of the solitary tract (NTS). Mechanistically, high glucose activated intrinsic excitability and the amplitude of spontaneous excitatory postsynaptic currents in NTS glutamatergic neurons; congruently, selective activation of NTS glutamatergic neurons mimicked anxiety-like behavior in mice, while chemogenetic silencing of these neurons abolished glucose-induced anxiety. Together, our findings demonstrate that NTS glutamatergic neurons activation mediates sugar-induced anxiety. These results suggest that this anxiogenic effect is driven by glucose-related signaling rather than artificial sweet taste perception alone, shedding light on a novel clinical therapy against anxiety.

## Simple Summary

High sugar intake is linked to anxiety, yet the underlying brain mechanisms remain poorly understood. This study investigated whether short-term consumption of sugar or artificial sweeteners induce anxiety, and which brain pathways mediate this effect. Experiments revealed that two weeks of sugar-sweetened water triggered anxiety in mice, while artificial sweeteners did not. To understand why this happened, the researchers identified that specific nerve cells in the brainstem, which normally monitor the body’s internal state, become hyperactive after sugar consumption. Experimental activation of these cells induced anxiety, while silencing them prevented sugar-induced anxiety. This reveals a direct brain pathway through which sugar impacts mood. The findings suggest that limiting consumption of sugary beverages may benefit mental health and could point to new targets for future anxiety treatments.

## 1. Introduction

The rapid industrialization of the global food supply and changing dietary patterns have contributed to a substantial increase in sugar consumption, representing a major public health challenge [1,2]. Beyond metabolic effects, accumulating evidence highlights its detrimental impact on the central nervous system and mental health, particularly in relation to the development of anxiety disorders [3,4,5,6,7,8]. However, the relationship is more complex than initially appreciated. Although epidemiological studies have reported associations between high-sugar dietary patterns and elevated anxiety risk in humans [9,10,11,12,13], inconsistencies persist, with some reporting no significant association or even inverse relationships [14,15,16]. Moreover, the observational design of this cross-sectional evidence limits causal inference, as reverse causation, whereby anxiety itself drives increased sugar consumption, and residual confounding (e.g., from overall dietary quality or socioeconomic status) cannot be excluded. In preclinical research, the evidence is similarly nuanced: while several rodent studies indicate that chronic high-sugar intake, especially during adolescence, can induce anxiety-like behaviors [17,18], others report anxiolytic or null effects depending on the context of exposure [19,20,21]. Notably, some anxiety-related effects appear to be driven not by sugar intake per se, but by its withdrawal; for instance, sucrose withdrawal has been shown to trigger anxiety-like states through profound neuroadaptations in reward-related circuits [22,23]. Despite accumulating interest, the underlying neural mechanisms linking dietary sugar to anxiety remain poorly understood.

Although previous research has shed light on the link between high-sugar diets and anxiety from a top-down perspective, focusing on limbic structures involved in emotional regulation, such as the hippocampus and frontal cortex, or on systemic consequences like neuroinflammation [18,24,25,26], these studies have largely overlooked potential pathways that relay peripheral metabolic signals to the brain. To fully understand these peripheral metabolic signals, it is important to distinguish between two primary direct neural routes through which sugar may influence brain function and behavior: the sensory experience of sweet taste, initiated by T1R2/T1R3 receptors in taste buds and relayed via the chorda tympani and thalamus to the insula and orbitofrontal cortex [27]; and the post-ingestive gut–brain axis pathway, in which intestinal glucose sensing via sodium-glucose cotransporter (SGLT) triggers vagal afferent activation independent of orosensory input [28,29]. While sweet taste perception is activated by any sweetener regardless of caloric content, the SGLT1-mediated pathway responds selectively to glucose and structurally related sugars and conveys metabolic information to the brain via the vagus nerve [30,31,32].

The nucleus of the solitary tract (NTS), the principal brainstem nucleus receiving interoceptive sensory input from the body, including metabolic information from the periphery via the vagus nerve, serves as a critical gateway for gut–brain communication [33,34]. Recent studies have shown that as the primary central relay for vagal afferents from the gastrointestinal tract, the NTS monitors peripheral nutrient status through its glucose-sensing neurons [31,32]. Moreover, the NTS functions as a key hub for integrating visceral sensory information with autonomic and stress responses. It forms robust connections with limbic structures such as the amygdala and the hypothalamic–pituitary–adrenal axis, making it a plausible candidate for translating a high-sugar diet into altered emotional states [35,36,37]. Within the NTS, glutamatergic neurons constitute the predominant excitatory subpopulation receiving vagal afferent input [38,39]. Because these neurons mediate direct projections to the anxiety-related circuits, they are well positioned to bridge peripheral metabolic signaling with anxiety-like states [40]. However, direct causal evidence linking the NTS glutamatergic neurons to high-sugar diet-induced anxiety remains lacking.

Here, by employing behavioral analysis, immunofluorescence, in vitro brain slice electrophysiology, and chemogenetic manipulations in mice, we demonstrate that a high-sugar diet induces anxiety-like behavior via the sugar molecule itself, rather than through its taste or metabolic products. This effect is mediated by the activation of NTS glutamatergic neurons, revealing a novel bottom-up neural axis that links the peripheral metabolic state to central emotional processing.

## 2. Materials and Methods

### 2.1. Animal Care and Use

The Laboratory Animal Welfare and Ethics Committee of Jinan University granted approval for all animal studies, which were carried out in compliance with the national rules for animal care in China (Approval No. IACUC-20211123-03). A total of 171 male C57BL/6 mice aged six to eight weeks were employed in this study. Only male mice were included to minimize estrous cycle-induced variability in anxiety-related behaviors in females [41,42,43]. The animals were housed in groups on a 12:12 light-dark schedule (lights on at 07:00–19:00, lights off at 19:00–07:00) with constant ambient thermal and humidity settings and ad libitum access to food and water. Mice were divided into experimental and control groups at random. After the start of the intervention, the experimental group received drinking water containing 300 mM sucrose, 600 mM D-glucose, 600 mM methyl-alpha-D-glucopyranoside (MDG), or 30 mM acesulfame potassium (Ace-K) in place of plain water. The concentrations were selected to provide comparable sweetness intensity across solutions: 300 mM sucrose was used as the reference sweet solution, a concentration that approximates the sugar content of common sugary beverages and is generally well accepted by mice [31]; 600 mM D-glucose and 600 mM MDG were chosen to match the sweetness of 300 mM sucrose [44], with 600 mM D-glucose providing a caloric content equivalent to that of 300 mM sucrose, whereas MDG served as a non-metabolizable analog to dissociate sweet taste signaling from post-ingestive metabolic effects; and 30 mM Ace-K, a non-nutritive sweetener, was selected to achieve similar sweetness intensity without caloric input or metabolic signaling, allowing further separation of pure taste effects from gut-derived signals [32]. Water bottles were replaced every three days to prevent microbial contamination. Experimenters blinded to group assignment performed the behavioral tests and data analysis. On a daily basis, the animals were observed for signs of acute suffering or poor general condition, and humanely euthanized if needed. Every attempt was made to employ fewer animals and to lessen their suffering.

### 2.2. Open Field Test

To conduct the open field test, individual mice were gently placed in the center of a square arena (40 cm × 40 cm × 40 cm) under uniform illumination. The mice were then allowed to roam freely for five minutes, during which time an overhead camera captured the activity. The entire arena was thoroughly cleaned with 70% ethanol in between trials to avoid olfactory cues, and the following video analysis using automated tracking software measured the total distance traveled as a measure of general locomotion as well as the distance traveled in the central zone (defined as the central 25% of the total area) to evaluate anxiety-like behavior. The animals were presented with the testing environment for at least one hour before the experiment, and all testing took place in quiet conditions during the light phase.

### 2.3. Elevated Plus Maze

The elevated plus-maze test consisted of two open arms and two enclosed arms of identical dimensions (10 cm × 50 cm), and the maze was elevated 50 cm above the ground. At the start of the test, each mouse was carefully placed with its head directed toward an open arm and given 5 min of free exploration. An overhead camera observed their activity, and an automated monitoring system quantified the time spent in the open arms to assess anxiety-like behavior. Between consecutive trials, the maze was wiped down with 70% ethanol to remove residual olfactory cues. The animals were presented with the testing environment for at least one hour before the experiment, and all testing took place in quiet conditions during the light phase.

### 2.4. Immunofluorescence

Mice were transcardially perfused with 0.9% saline and then 4% paraformaldehyde (PFA) after being thoroughly anesthetized with Avertin (13 μL/g, i.p.) for immunofluorescence. After carefully removing the brains, they were post-fixed in a 4% PFA solution at 4 °C for the entire night before being cryoprotected in solutions of 20% and 30% sucrose until they sank sequentially. A freezing microtome (Leica CM1950, Leica Biosystems, Wetzlar, Germany) was used to create coronal slices (40 μm thick) including the NTS. Slices were blocked for one hour at room temperature in a blocking solution comprising 5% normal goat serum (NGS) and 0.3% Triton X-100 in PBS to permeabilize membranes and prevent non-specific binding following three rounds of washing with 0.01 M phosphate-buffered saline (PBS). After that, the slices were incubated for 24 h at 4 °C with the following primary antibody cocktails diluted in blocking solution: mouse anti-CaMKII (1:500, Cell Signalling Technology, cat # 50049, Danvers, MA, USA) to label glutamatergic neurones, rabbit anti-c-Fos (1:500, Cell Signalling Technology, cat # 2250, Danvers, MA, USA) to label activated neurones, and chicken anti-GAD67 (1:500, Abcam, cat # ab75712, Cambridge, UK) to label GABAergic neurones. Donkey anti-mouse IgG conjugated with Alexa Fluor 488 (1:500, Jackson ImmunoResearch, cat# 715-545-150, West Grove, PA, USA) for CaMKII, donkey anti-chicken IgG conjugated with Alexa Fluor 488 (1:500, Jackson ImmunoResearch, cat# 703-545-155, West Grove, PA, USA) for GAD67, and donkey anti-rabbit IgG conjugated with Cy3 (1:500, Jackson ImmunoResearch, cat# 711-165-152, West Grove, PA, USA). Following final PBS washes, slices were counterstained with DAPI (1:1000) for 10 min to see cell nuclei. Using an anti-fade mounting medium, each slice was placed onto a glass slide and coverslipped. A confocal laser scanning microscope (Zeiss LSM 880, Carl Zeiss AG, Oberkochen, Germany) with a 20× objective was used to capture the images. Three to four slices from the area containing NTS were photographed for each animal using the same laser power and gain settings. An investigator who was blind to the group assignment counted c-Fos-positive nuclei (red) and CaMKII or GAD67-positive cells (green) either manually or with image analysis software (ImageJ 1.54g).

### 2.5. Virus Injection

Following induction of deep anesthesia with Avertin (13 μL/g, i.p.), mice were secured in a stereotaxic frame (RWD Life Science, Shenzhen, China). Viral vectors were then subsequently pressure-injected into the NTS (AP: −7.4 mm; ML: ±0.5 mm; DV: −3.9 mm) at 0.1 μL/min through a glass micropipette (Microinjection Syringe Pump, WPI, Sarasota, FL, USA). The pipette remained in position for roughly 5 min following injection to facilitate diffusion. Post-wound closure, topical antibiotics were used, and ketoprofen (5 mg/kg) was administered subcutaneously for postoperative analgesia, following which the mice recuperated under a heat lamp.

### 2.6. Electrophysiology

Acute brain slices were prepared following established protocols [45]. Briefly, mice were thoroughly anesthetized with isoflurane and subsequently decapitated. The brain was swiftly excised and submerged in an ice-cold, oxygenated cutting solution (95% O_2_/5% CO_2_) comprising (in mM): 119 NaCl, 26.2 NaHCO_3_, 11 D-glucose, 2.5 KCl, 1 NaH_2_PO_4_, 2.5 CaCl_2_, and 1.3 MgCl_2_, at pH 7.4 and 290 mOsm. Coronal brain slices (250 μm thick) encompassing the NTS were prepared utilizing a vibratome (VT1200S; Leica Biosystems, Wetzlar, Germany) and were promptly transferred to a holding chamber containing standard artificial cerebrospinal fluid (ACSF) composed of (in mM): 126 NaCl, 26 NaHCO_3_, 10 D-glucose, 3 KCl, 1.25 NaH_2_PO_4_, 2 CaCl_2_, and 1 MgSO_4_. The ACSF was constantly pumped with the mixed gas (95% O_2_/5% CO_2_). Brain slices were incubated at 34 °C for 30 min to allow recovery, then maintained at ambient temperature for minimum of 1 h prior to electrophysiological recording.

For recording, individual brain slices were carefully transferred to a recording chamber, where they were continuously superfused with oxygenated ACSF at a constant perfusion rate of 2–3 mL/min to maintain tissue viability and stable physiological conditions. Neurons in the NTS region were observed utilizing an infrared differential interference contrast (IR-DIC) microscope (FN1, Nikon, Tokyo, Japan) fitted with a 40× water-immersion objective. Epifluorescence illumination was employed with eGFP and mCherry filter sets to detect transfected neurons, and images were acquired using a digital camera (C11440, Hamamatsu Photonics K.K., Hamamatsu, Japan). Patch pipettes (4–7 MΩ) were constructed from borosilicate glass capillaries and filled with an internal solution comprising (in mM): 140 K-gluconate, 4.5 MgCl_2_, 5 EGTA, 9 HEPES, 4 MgATP, 4.4 Na_2_-phosphocreatine and 0.3 Na_3_GTP (290 mOsm, pH adjusted to 7.4 with KOH). Once a GΩ seal was achieved, a short pulse of gentle negative pressure was applied to rupture the patch membrane and establish the whole-cell voltage-clamp configuration. Series resistance (Rs) was tracked in real time throughout the recording duration and adjusted by 70–80% to reduce voltage mistakes. If the series resistance was more than 20 MΩ or the fluctuation was more than 20% of the baseline value, the recordings were discarded and not included in further analysis. An EPC-10 patch-clamp amplifier (HEKA Elektronik, Lambrecht, Germany) was used to amplify all electrophysiological signals, which were then digitally recorded using PatchMaster software (version 2.73).

Neurons were voltage-clamped at a holding potential of −70 mV to record spontaneous excitatory postsynaptic currents (sEPSCs), with data acquisition performed continuously over a 2 min period for each cell. To assess intrinsic excitability, neurons were voltage-clamped at a holding potential of −70 mV. To evoke action potentials, two stimulation protocols were applied under current-clamp mode: a set of depolarizing current pulses with 500 ms duration, delivered in 10-pA increments from 0 up to 150 pA; and a linear depolarizing current ramp ranging from 0 to 160 pA applied over 800 ms. The efficacy of chemogenetic viruses was tested by measuring action potential firing in identified hM4Di-eGFP- or hM3Dq-mCherry-expressing neurons, before and after perfusion with 5 μM clozapine-N-oxide (CNO), using identical depolarizing current injections.

### 2.7. Chemogenetic Manipulation

For chemogenetic inhibition or activation of CaMKII-expressing neurons in NTS, recombinant adeno-associated viruses (AAV2/9) were bilaterally microinjected into the NTS. Control groups received AAV2/9-CaMKII-eGFP-WPRE-pA or AAV2/9-CaMKII-mCherry-WPRE-pA, while experimental groups were injected with AAV2/9-CaMKII-hM4Di-eGFP (for inhibition) or AAV2/9-CaMKII-hM3Dq-mCherry (for activation). All viral vectors were prepared at a titer of 1.0 × 10^13^ GC/mL, with a volume of 0.08 μL per hemisphere (TaiTool Bioscience, Shanghai, China). To achieve chemogenetic manipulations in vivo, CNO at 1 mg/kg was injected intraperitoneally injection 30 min before the start of behavior tests.

### 2.8. Statistical Analysis

We used GraphPad Prism (version 9.0) for all our statistical analysis in this study. The mean ± standard error of the mean (SEM) is used to express the data. For behavioral experiments, n represents the number of mice per group. For immunofluorescence analysis, n represents the number of brain slices analyzed, with multiple slices collected from each mouse. For electrophysiological recordings, n represents the number of neurons recorded, with multiple neurons examined from each mouse. Prior to parametric analysis, the Shapiro–Wilk test was used to determine the normality of the data distribution, and Levene’s test was applied to evaluate homoscedasticity. An unpaired or paired two-tailed Student’s *t*-test was used, depending on the situation, to compare two groups. One-way or two-way repeated measures ANOVA was used for comparisons between more than two groups, and Fisher’s LSD test was adopted for subsequent pairwise comparisons. Differences were regarded as statistically significant when *p* < 0.05.

### 2.9. Generative Artificial Intelligence Tool Use

During the preparation of this manuscript, the generative AI tool GPT-4o (OpenAI) was employed to assist with language polishing, grammatical correction, and improvement of readability. All scientific content, data interpretation, and conclusions were independently developed and finalized by the authors.

## 3. Results

### 3.1. Two Weeks, but Not One Week, of Sucrose Consumption Induced Anxiety-Like Behavior in Mice

**Figure 1:**
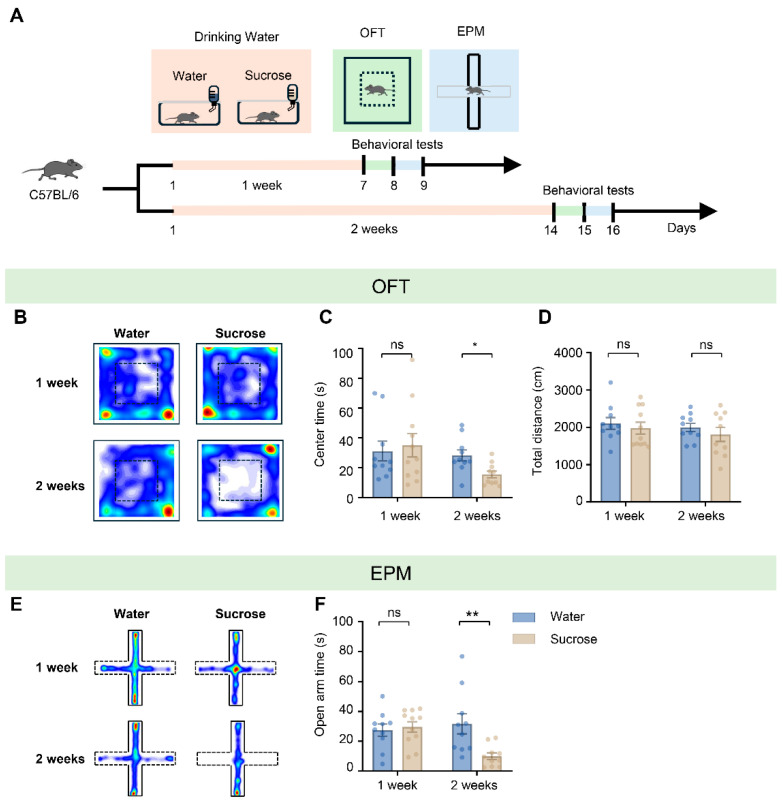
Two weeks of sucrose treatment induced anxiety-like behavior in mice. (**A**) Schematic of experimental design and behavioral assessments. C57BL/6 mice were assigned to water or sucrose treatment groups for durations of 1 week or 2 weeks. Behavioral assessments were conducted using the open field test (OFT) and the elevated plus maze (EPM) following the respective treatment periods. (**B**) Representative heatmaps of locomotor trajectories for different treatment groups in the OFT. (**C**,**D**) Statistical analysis of center time (**C**) and total distance traveled (**D**) in the OFT (Unpaired Student’s *t*-test, center time: 1 week, *p* = 0.709, 2 weeks, *p* = 0.010; total distance: 1 week, *p* = 0.574, 2 weeks, *p* = 0.400; *n* = 10–11 mice per group). (**E**) Representative heatmaps of locomotor trajectories for different treatment groups in the EPM test. (**F**) Statistical analysis of open arm time in the EPM (Unpaired Student’s *t*-test, 1 week, *p* = 0.699, 2 weeks, *p* = 0.007, *n* = 10–11 mice per group). Data are presented as mean ± SEM; * *p* < 0.05, ** *p* < 0.01, and “ns” indicates no significant difference.

To determine whether a brief period of high-sugar intake is sufficient to induce anxiety-like behaviors, C57BL/6 mice were provided with 300 mM sucrose water (a concentration chosen to mimic the sugar content of typical sugary beverages) and divided into two groups receiving sucrose for one week or two weeks, respectively (Figure 1A). Anxiety-like behavior was evaluated using the OFT and the EPM tests [46]. Mice exposed to sucrose for 1 week showed no significant differences in OFT measures relative to the water-control group (center time: water group = 31.05 ± 6.63 s, sucrose group = 34.99 ± 7.88 s; water vs. sucrose, *t* = 0.378, *df* = 19, *p* = 0.709; total distance: water group = 2106.00 ± 157.28 cm, sucrose group = 1978.94 ± 156.44 cm; water vs. sucrose, *t* = 0.572, *df* = 19, *p* = 0.574; *n* = 10 for water, *n* = 11 for sucrose; Figure 1B–D). In contrast, after 2 weeks of sucrose exposure, mice spent significantly less time exploring the center zone without a reduction in total travel distance (center time: water group = 28.20 ± 3.77 s, sucrose group = 15.46 ± 2.35 s; water vs. sucrose, *t* = 2.87, *df* = 18, *p* = 0.010; total distance: water group = 1996.85 ± 112.97 cm, sucrose group = 1809.04 ± 186.04 cm; water vs. sucrose, *t* = 0.863, *df* = 18, *p* = 0.400; *n* = 10 mice per group; Figure 1B–D). Consistent results were obtained in the EPM: mice exposed to sucrose for 2 weeks, but not 1 week, displayed a significant decrease in the open-arm exploration time (1 week: water group = 27.88 ± 4.07 s, *n* = 10 mice, sucrose group = 29.59 ± 3.56 s, *n* = 11 mice; water vs. sucrose, *t* = 0.393, *df* = 19, *p* = 0.699; 2 week: water group = 31.75 ± 6.83 s, sucrose group = 9.96 ± 2.27 s, *n* = 10 mice per group; water vs. sucrose, *t* = 3.027, *df* = 18, *p* = 0.007; Figure 1E,F). Together, these findings indicate that 2 weeks, but not 1 week, of sucrose consumption elicits anxiety-like behavior in mice. We also measured changes in water bottle weight to calculate the daily fluid intake in the 2 weeks exposed group and found a 22% increase in fluid consumption compared to water-control group (Supplemental Figure S1), confirming the mice were adequately exposed to sucrose and exhibited a preference for it.

### 3.2. Sugar-Induced Anxiety-Like Behavior Involves Glucose-Like Molecular Structure Beyond Sweet Taste

**Figure 2:**
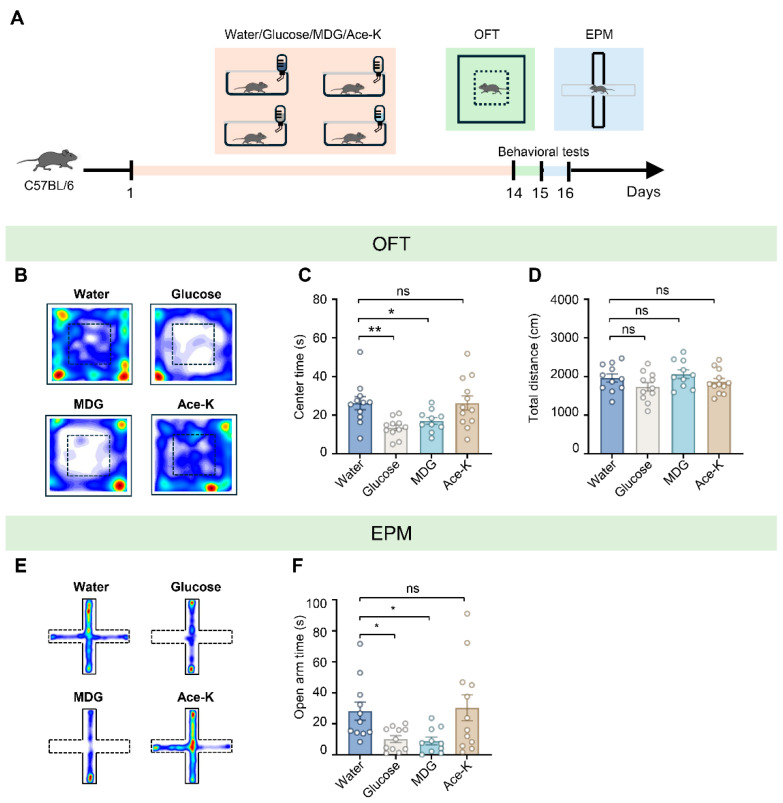
Sucrose, glucose, and MDG, but not Ace-K, induce anxiety-like behavior. (**A**) Schematic timeline of experimental design and behavioral assessments. C57BL/6 mice were administered water, glucose, MDG (non-metabolizable glucose analog), or Ace-K (non-nutritive sweetener) for 14 consecutive days. Behavior was evaluated using the open field test and elevated plus maze (EPM) on days 15 and 16 respectively. (**B**) Representative heatmaps of locomotor trajectories for four different treatment groups in the OFT. (**C**,**D**) Statistical analysis of center time (**C**) and total distance traveled (**D**) in the OFT (One-way ANOVA, center time: water vs. glucose, *p* = 0.003, water vs. MDG, *p* = 0.034, water vs. Ace-K, *p* = 0.991; total distance: water vs. glucose, *p* = 0.133, water vs. MDG, *p* = 0.506, water vs. Ace-K, *p* = 0.509; *n* = 10–12 mice per group). (**E**) Representative heatmaps of locomotor trajectories for four different treatment groups in the EPM. (**F**) Statistical analysis of open arm time in the EPM (One-way ANOVA, Water vs. Glucose, *p* = 0.030, Water vs. MDG, *p* = 0.024, Water vs. Ace-K, *p* = 0.786, *n* = 10–12 mice per group). Data are presented as mean ± SEM; * *p* < 0.05, ** *p* < 0.01, and “ns” indicates no significant difference.

Having shown that sucrose induces anxiety-like behavior, we next asked whether this effect relies on sweet taste perception alone or additionally requires molecular sensing of glucose-like structure, distinct from its metabolic breakdown. To address this, we compared the effects of a 2-week exposure to different sweet solutions, including caloric glucose solution, non-metabolizable glucose analog MDG, and a non-nutritive sweetener Ace-K (Figure 2A). Consistent with the sucrose findings, 2-week 600 mM D-glucose consumption produced significant anxiety-like behavior. In the OFT, these mice spent significantly less time exploring the center zone, without a reduction in total travel distance compared to the water-control group (center time: water group = 26.20 ± 3.40 s, glucose group = 13.52 ± 1.49 s; water vs. glucose, *t* = 3.130, *df* = 40, *p* = 0.003; total distance: water group = 1960.37 ± 106.18 cm, glucose group = 1734.02 ± 112.44 cm; water vs. glucose, *t* = 1.53, *df* = 40, *p* = 0.133; *n* = 11 for water, *n* = 11 for glucose; Figure 2B–D). This anxiogenic effect was corroborated by EPM, where the glucose group spent significantly less time in the open arms (water group = 28.22 ± 5.89 s, glucose group = 10.20 ± 2.26 s; water vs. glucose, *t* = 2.248, *df* = 40, *p* = 0.030; *n* = 11 for water, *n* = 11 for glucose; Figure 2E,F). Notably, exposure to MDG, which provides sweet taste but cannot be metabolized for energy, recapitulated the anxiogenic effect to a similar extent as glucose (center time: MDG group = 17.11 ± 1.76 s; water vs. MDG, *t* = 2.191, *df* = 40, *p* = 0.034; total distance: MDG group = 2061.97 ± 111.96 cm; water vs. MDG, *t* = 0.670, *df* = 40, *p* = 0.506; open arm time: MDG group = 8.96 ± 2.53 s; water vs. MDG, *t* = 2.344, *df* = 40, *p* = 0.024; *n* = 10 for MDG; Figure 2B–F). In contrast, mice exposed to Ace-K showed no significant differences in either test compared to controls (center time: Ace-K group = 26.25 ± 3.71 s; water vs. Ace-K, *t* = 0.011, *df* = 40, *p* = 0.991; total distance: Ace-K group = 1863.99 ± 89.32 cm; water vs. Ace-K, *t* = 0.666, *df* = 40, *p* = 0.509; open arm time: Ace-K group = 30.37 ± 8.34 s; water vs. Ace-K, *t* = 0.273, *df* = 40, *p* = 0.786; *n* = 12 for Ace-K; Figure 2B–F). These data suggest that the anxiogenic effect likely extends beyond sweet taste perception alone, given that the non-nutritive sweetener Ace-K failed to induce anxiety. Instead, the shared chemical structure of glucose and MDG, or the activation of specific physiological pathways, appears to be critical for driving this behavioral response.

### 3.3. Two Weeks of Sugary Drinks Consumption Activates c-Fos Expression of the Nucleus of the Solitary Tract Especially in CaMKII Positive Neurons

**Figure 3:**
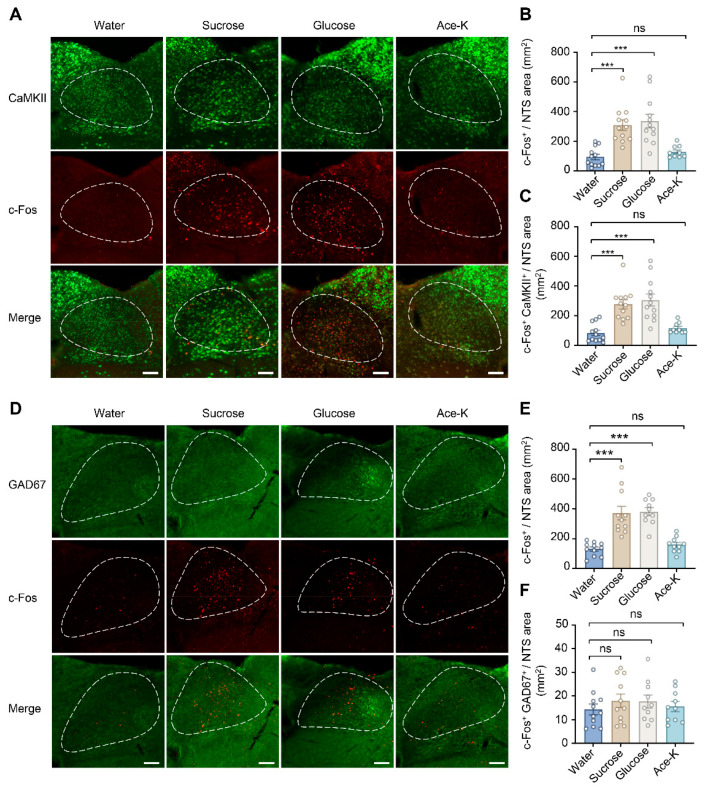
Two weeks of sugary drink treatment activated c-Fos expression in CaMKII-positive neurons of the nucleus of the solitary tract. (**A**) Representative images of immunofluorescence staining for CaMKII and c-Fos in different treatment groups. Scale bar: 100 µm. (**B**,**C**) Density of c-Fos-positive cells (**B**) and cells co-labeled for c-Fos and CaMKII (**C**) in the nucleus of the solitary tract (NTS) brain region (One-way ANOVA, c-Fos^+^: water vs. glucose, *p* < 0.001, water vs. glucose, *p* < 0.001, water vs. Ace-K, *p* = 0.483; c-Fos^+^+CaMKII^+^: water vs. glucose, *p* < 0.001, water vs. glucose, *p* < 0.001, water vs. Ace-K, *p* = 0.465; *n* = 9–12 slices from 3 to 4 mice per group). (**D**) Representative images of immunofluorescence staining for GAD67 and c-Fos in different treatment groups. Scale bar: 100 µm. (**E**,**F**) Density of c-Fos-positive cells (**E**) and cells co-labeled for c-Fos and GAD67 (**F**) in the NTS brain region (One-way ANOVA, c-Fos^+^: water vs. glucose, *p* < 0.001, water vs. glucose, *p* < 0.001, water vs. Ace-K, *p* = 0.490; c-Fos^+^+GAD67^+^: water vs. glucose, *p* = 0.312, water vs. glucose, *p* = 0.351, water vs. Ace-K, *p* = 0.734; *n* = 10–11 slices from 4 mice per group). Data are presented as mean ± SEM; *** *p* < 0.001, “ns” indicates no significant difference.

A previous study reported that NTS is selective for transducing gut–brain sugar signals, showing a preference for glucose over Ace-K [32]. To further investigate changes in the activity of two major types of neurons in NTS following high sugar exposure, we used c-Fos, CaMKII, and GAD67 as markers for labeling neuronal activation, glutamatergic neurons, and GABAergic neurons, respectively, in mice that consumed sugary drinks for 2 weeks (Figure 3A,D) [47]. Compared with the water-control group, the number of c-Fos-positive neurons significantly increased in the NTS of mice exposed to sucrose and glucose, whereas no change was observed in the Ace-K group (CaMKII: water group = 93.41 ± 18.69, sucrose group = 309.05 ± 36.46, glucose group = 336.35 ± 46.02, Ace-K group = 128.40 ± 13.56; water vs. sucrose, *t* = 4.789, *df* = 41, *p* < 0.001; water vs. glucose, *t* = 5.479, *df* = 41, *p* < 0.001; water vs. Ace-K, *t* = 0.738, *df* = 41, *p* = 0.465; *n* = 12 slices from 4 mice for water, sucrose and glucose, *n* = 9 slices from 3 mice for Ace-K; GAD67: water group = 130.69 ± 13.61, sucrose group = 372.16 ± 45.47 glucose group = 380.69 ± 26.95, Ace-K group = 159.38 ± 16.25; water vs. sucrose, *t* = 1.025, *df* = 38, *p* = 0.312; water vs. glucose, *t* = 0.944, *df* = 38, *p* = 0.351; water vs. Ace-K, *t* = 0.342, *df* = 38, *p* = 0.734; *n* = 11 slices from 4 mice for water and sucrose, *n* = 10 slices from 4 mice for glucose and Ace-K; Figure 3B,E). This indicates that 2 weeks of sugar consumption activates neuronal activity in the NTS, while Ace-K does not. Moreover, the activity of glutamatergic neurons in the NTS, reflected by increased numbers of c-Fos and CaMKII double-positive neurons, was also enhanced in the sucrose and glucose groups relative to the water-control group, but not in the Ace-K group (water group = 83.18 ± 17.90, sucrose group = 276.60 ± 30.85, glucose group = 304.49 ± 41.12, Ace-K group = 115.38 ± 11.96; water vs. sucrose, *t* = 4.716, *df* = 41, *p* < 0.001; water vs. glucose, *t* = 5.313, *df* = 41, *p* < 0.001; water vs. Ace-K, *t* = 0.708, *df* = 41, *p* = 0.483; *n* = 12 slices from 4 mice for water, sucrose and glucose, *n* = 9 slices from 3 mice for Ace-K; Figure 3C). In contrast, there was no difference among the four groups in the numbers of c-Fos and GAD67 double-positive neurons (water group = 14.32 ± 2.30, sucrose group = 17.91 ± 2.85, glucose group = 17.70 ± 2.75, Ace-K group = 15.55 ± 2.10; water vs. sucrose, *t* = 6.014, *df* = 38, *p* < 0.001; water vs. glucose, *t* = 6.077, *df* = 38, *p* < 0.001; water vs. Ace-K, *t* = 0.697, *df* = 38, *p* = 0.490; *n* = 11 slices from 4 mice for water and sucrose, *n* = 10 slices from 4 mice for glucose and Ace-K; Figure 3F). These results suggest that the neuronal activation in the NTS driven by sugar consumption occurs primarily in glutamatergic neurons.

### 3.4. Electrophysiological Alterations of Glutamatergic Neurons in the Nucleus of the Solitary Tract Following Two-Week Consumption of Glucose Drinks

**Figure 4:**
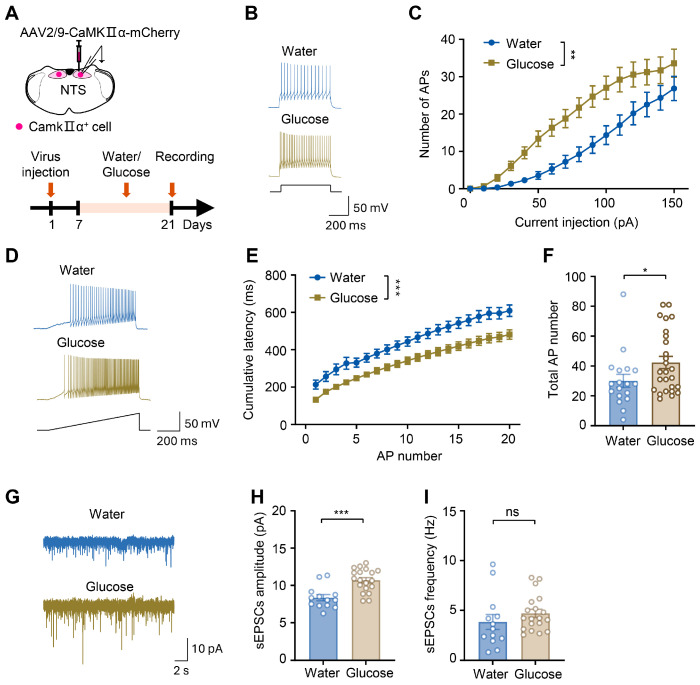
Electrophysiological alterations of glutamatergic neurons in the nucleus of the solitary tract following two-week consumption of glucose drinks. (**A**) Schematic of virus injection and electrophysiological recording timeline. AAV2/9-CaMKIIα-mCherry was stereotactically injected into the nucleus of the solitary tract (NTS) to label CaMKIIα-positive glutamatergic neurons. Following viral expression, mice received either water or glucose treatment for 2 weeks. Whole-cell patch-clamp recordings were performed in labeled neurons on day 21. (**B**,**D**) Representative action potential (AP) traces of NTS glutamatergic neurons following water or glucose treatment. (**C**) The number of action potentials in NTS glutamatergic neurons from water- and glucose-treated mice against depolarizing currents (Two-way repeated measures ANOVA, *p* = 0.007, *n* = 18–26 neurons from 3 to 4 mice per group). (**E**,**F**) Action potential latency (**E**) and total number of action potentials (**F**) in NTS glutamatergic neurons under depolarizing current ramps (Two-way repeated measures ANOVA and unpaired Student’s *t*-test, latency: *p* < 0.001; AP number: *p* = 0.049; *n* = 18–26 neurons from 3 to 4 mice from per group). (**G**) Representative traces of spontaneous excitatory postsynaptic currents (sEPSCs) recorded from NTS glutamatergic neurons in water- or glucose-treated mice. (**H**,**I**) Amplitude (**H**) and frequency (**I**) of sEPSCs in NTS glutamatergic neurons of mice treated with water or glucose (Unpaired Student’s *t*-test, amplitude: *p* < 0.001, frequency: *p* = 0.288, *n* = 13–19 neurons from 3 mice per group). Data are presented as mean ± SEM; * *p* < 0.05, ** *p* < 0.01, *** *p* < 0.001, and “ns” indicates no significant difference.

To further validate the functional alterations of NTS glutamatergic neurons in high-sugar-exposed mice, electrophysiological recordings were conducted in brain slices. Virus AAV2/9-CaMKIIα-mCherry was injected into the NTS to label glutamatergic neurons, and following at least 3 weeks of viral expression post-injection, we performed whole-cell clamp-patch recordings in neurons from mice exposed to 2 weeks of 600 mM glucose in their drinking water or a water-control (Figure 4A). To assess the intrinsic excitability of NTS glutamatergic neurons, we recorded their responses to graded depolarizing current injections. Representative action potential (AP) traces showed denser AP firing in neurons from glucose-treated mice compared to water controls (Figure 4B). The input-output relationship further confirmed that the number of evoked APs was significantly higher in the glucose group across increasing current injection intensities (water group: *n* = 18 neurons from 3 mice, glucose group: *n* = 26 neurons from 4 mice; water vs. glucose, *F* (1, 42) = 7.948, *df* = 1, *p* = 0.007; Figure 4C). Neuronal excitability was further quantified by determining both the AP onset latency and the total number of spikes generated in response to depolarizing current ramp injections. (Figure 4D). Similarly, 2 weeks of glucose exposure significantly reduced the AP latency (water group: *n* = 18 neurons from 3 mice, glucose group: *n* = 26 neurons from 4 mice; water vs. glucose, *F* (1, 30) = 14.20, *df* = 1, *p* < 0.001, *n* = 18–26 neurons from 3 to 4 mice per group; Figure 4E) and increased the total number of evoked APs (water group = 30.06 ± 4.32, *n* = 18 neurons from 3 mice, glucose group = 42.38 ± 4.08, *n* = 26 neurons from 4 mice; water vs. glucose, *t* = 2.027, *df* = 42, *p* = 0.049; Figure 4F). To assess synaptic function in these neurons, we recorded spontaneous excitatory postsynaptic currents (sEPSCs). Representative traces and quantitative analysis are shown in Figure 4G. In glucose-exposed mice, sEPSC amplitude was significantly increased (water group = 8.36 ± 0.41 pA, *n* = 13 neurons from 3 mice, glucose group = 10.69 ± 0.35 pA, *n* = 19 neurons from 3 mice; water vs. glucose, *t* = 4.247, *df* = 30, *p* < 0.001; Figure 4H), whereas the frequency remained unchanged (water group = 3.83 ± 0.75 Hz, *n* = 13 neurons from 3 mice, glucose group = 4.69 ± 0.41 Hz, *n* = 19 neurons from 3 mice; water vs. glucose, *t* = 1.082, *df* = 30, *p* = 0.288; Figure 4I). This pattern indicates that glucose exposure enhances postsynaptic responsiveness without affecting the presynaptic release probability. Collectively, these data demonstrate that glucose consumption potentiates both the intrinsic excitability and synaptic strength of NTS glutamatergic neurons.

### 3.5. Suppression or Activation Glutamatergic Neurons in the Nucleus of the Solitary Tract Bidirectionally Modulate Murine Anxiety-Like Behavior

**Figure 5:**
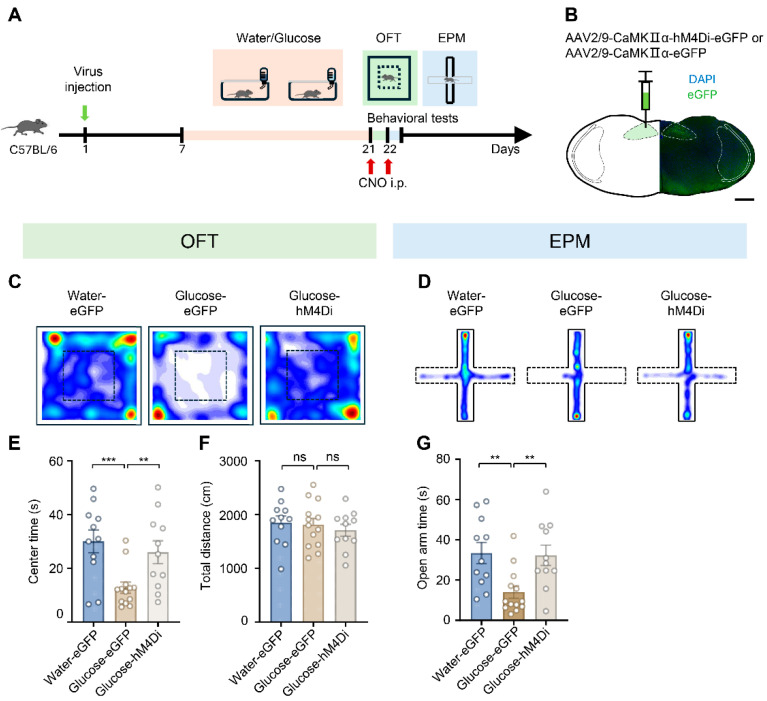
Inhibiting the nucleus of the solitary tract glutamatergic neurons abolishes glucose-induced anxiety-like behavior in mice. (**A**) Schematic timeline of chemogenetic inhibition and behavioral testing. C57BL/6 mice were injected with virus targeting the nucleus of the solitary tract (NTS) glutamatergic neurons. Following a recovery and expression period, mice received water or glucose treatment for 2 weeks. On days 21 and 22, clozapine N-oxide (CNO) was intraperitoneally administered 30 min prior to the open field test (OFT) and elevated plus maze (EPM), respectively. (**B**) Representative fluorescence image showing expression of AAV2/9-CaMKIIα-hM4Di-eGFP (or control AAV2/9-CaMKIIα-eGFP) in the NTS. eGFP signal (green) indicates infected CaMKIIα-expressing glutamatergic neurons. Nuclei are counterstained with DAPI (blue). Scale bar: 500 μm. (**C**) Representative heatmaps of locomotor trajectories for different treatment groups in the OFT. (**D**) Representative heatmaps of locomotor trajectories for different treatment groups in the EPM. (**E**,**F**) Statistical analysis of center time (**E**) and total distance traveled (**F**) in the OFT (Unpaired Student’s *t*-test, center time: water-eGFP vs. glucose-eGFP, *p* < 0.001, glucose-eGFP vs. glucose-hM4Di, *p* = 0.008; total distance: water-eGFP vs. glucose-eGFP, *p* = 0.822, glucose-eGFP vs. glucose-hM4Di, *p* = 0.540; *n* = 11–13 mice per group). (**G**) Statistical analysis of open arm time in the EPM (Unpaired Student’s *t*-test, water-eGFP vs. glucose-eGFP, *p* = 0.003, glucose-eGFP vs. glucose-hM4Di, *p* = 0.004, *n* = 11–13 mice per group). Data are presented as mean ± SEM; ** *p* < 0.01, *** *p* < 0.001, and “ns” indicates no significant difference.

Based on the above results, 2 weeks of high-glucose exposure induces robust hyperactivity of NTS glutamatergic neurons. To test whether this hyperactivity is causally linked to glucose-induced anxiety-like behavior, we established three groups (Figure 5A): high-glucose-exposed mice injected with rAAV2/9-CaMKII-hM4Di-eGFP (to inhibit NTS glutamatergic neurons), high-glucose-exposed mice injected with control virus (rAAV2/9-CaMKII-eGFP, as high-glucose control), and water-fed mice injected with control virus (water-eGFP group, as normal behavior baseline) (Figure 5A). Specifically, we injected the respective viruses into the NTS (Figure 5B). Three weeks post-injection, whole-cell patch-clamp recordings in acute NTS slices demonstrated that the hM4Di agonist CNO (clozapine-N-oxide) effectively suppressed action potential firing in hM4Di-expressing neurons (Supplemental Figure S2A,B), confirming the efficacy of the chemogenetic approach. Following this, behavioral tests were conducted, and we found that chemogenetic suppression of the hyperactivity of NTS glutamatergic neurons rescued the mice from anxiety-like behavior induced by glucose exposure. In the OFT, the Glucose-hM4Di group showed increased center time compared to the glucose-eGFP group, returning to levels like the water-eGFP controls (water-eGFP group = 30.06 ± 4.25 s, *n* = 11 mice, glucose-eGFP group = 12.81 ± 2.07 s, *n* = 13 mice, glucose-hM4Di group = 26.00 ± 4.25 s, *n* = 11 mice; water-eGFP vs. glucose-eGFP, *t* = 3.834, *df* = 22, *p* < 0.001; glucose-eGFP vs. glucose-hM4Di, *t* = 2.928, *df* = 22, *p* = 0.008; Figure 5C,E). This rescue was confirmed in the EPM, where the glucose-4D group spent more time in the open arms than the glucose-eGFP group (water-eGFP group = 33.37 ± 5.24 s, *n* = 11 mice, glucose-eGFP group = 14.01 ± 3.12 s, *n* = 13 mice, glucose-hM4Di group = 32.31 ± 5.05 s, *n* = 11 mice; water-eGFP vs. glucose-eGFP, *t* = 3.291, *df* = 22, *p* = 0.003; glucose-eGFP vs. glucose-hM4Di, *t* = 3.183, *df* = 22, *p* = 0.004; Figure 5D,G). Meanwhile, locomotor activity (total distance) in OFT was unaffected (water-eGFP group = 1848.16 ± 124.56 cm, *n* = 11 mice, glucose-eGFP group = 1808.87 ± 118.09 cm, *n* = 13 mice, glucose-hM4Di group = 1707.33 ± 109.23 cm, *n* = 11 mice; water-eGFP vs. glucose-eGFP, *t* = 0.228, *df* = 22, *p* = 0.822; glucose-eGFP vs. glucose-hM4Di, *t* = 0.623, *df* = 22, *p* = 0.540; Figure 5F). These results demonstrate that inhibiting NTS glutamatergic neuron activity mitigates glucose-induced anxiety-like behavior.

**Figure 6:**
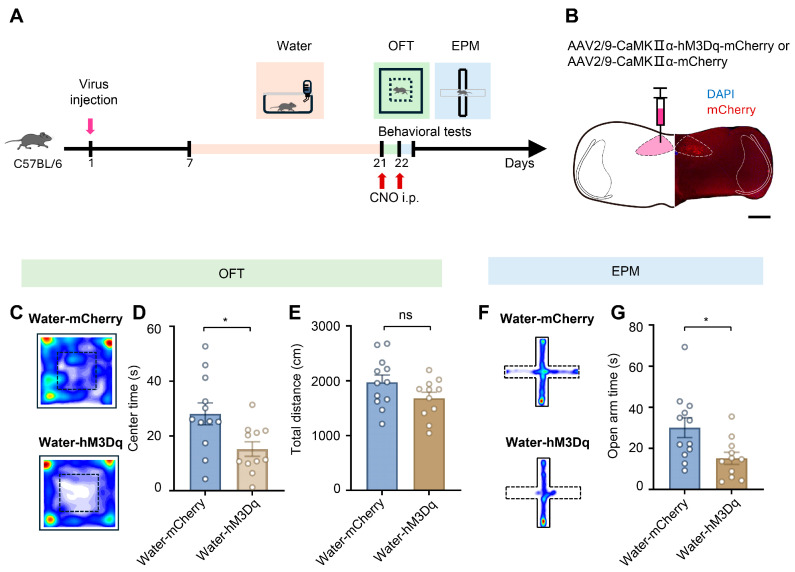
Activating the nucleus of the solitary tract glutamatergic neurons induces anxiety-like behavior in mice. (**A**) Schematic timeline of chemogenetic activation and behavioral testing. C57BL/6 mice were injected with virus targeting the nucleus of the solitary tract (NTS) glutamatergic neurons. Following a recovery and expression period, all mice received water treatment for 2 weeks. On days 21 and 22, clozapine N-oxide (CNO) was intraperitoneally administered 30 min prior to the open field test (OFT) and elevated plus maze (EPM), respectively. (**B**) Representative fluorescence image showing expression of AAV2/9-CaMKIIα-hM3Dq-mCherry (or control AAV2/9-CaMKIIα-mCherry) in the NTS. mCherry signal (red) indicates infected CaMKIIα-expressing glutamatergic neurons. Nuclei are counterstained with DAPI (blue). Scale bar: 500 μm. (**C**) Representative heatmaps of locomotor trajectories in the OFT for mice injected with hM3Dq virus versus control mice. (**D**,**E**) Statistical analysis of center time (**D**) and total distance traveled (**E**) in the OFT for mice injected with hM3Dq virus versus control mice. (Unpaired Student’s *t*-test, center time: *p* = 0.016; total distance: *p* = 0.105; *n* = 11–12 mice per group). (**F**) Representative heatmaps of locomotor trajectories in the EPM test for mice injected with hM3Dq virus versus control mice. (**G**) Statistical analysis of open arm time in the EPM test for mice injected with hM3Dq virus versus control mice. (Unpaired Student’s *t*-test, *p* = 0.017, *n* = 11–12 mice per group). Data are presented as mean ± SEM; * *p* < 0.05, “ns” indicates no significant difference.

To further determine whether activation of NTS glutamatergic neurons is sufficient to induce anxiety-like behavior, we expressed the excitatory DREADD hM3Dq (a Gq-coupled excitatory designer receptor) or a control virus in the NTS of mice (Figure 6A,B, Supplemental Figure S2C,D). Chemogenetic activation (hM3Dq group) in these neurons significantly reduced open arm time in the EPM (water-mCherry group = 30.07 ± 4.81 s, *n* = 12 mice, water-hM3Dq group = 15.16 ± 2.92 s, *n* = 11 mice; water-mCherry vs. water-hM3Dq, *t* = 2.59, *df* = 21, *p* = 0.017; Figure 6F,G) and center time in the OFT (water-mCherry group = 28.06 ± 4.01 s, *n* = 12 mice, water-hM3Dq group = 15.21 ± 2.64 s, *n* = 11 mice; water-mCherry vs. water-hM3Dq, *t* = 2.625, *df* = 21, *p* = 0.016; Figure 6C,D), without altering locomotor activity (water-mCherry group = 1972.71 ± 132.07 cm, *n* = 12 mice, water-hM3Dq group = 1680.46 ± 108.26 cm, *n* = 11 mice; water-mCherry vs. water-hM3Dq, *t* = 1.693, *df* = 21, *p* = 0.105; Figure 6E). These findings indicate that specific activation of NTS glutamatergic neurons is sufficient to induce anxiety-like behavior. Together, our chemogenetic inhibition and activation experiments show that NTS glutamatergic neurons bidirectionally regulate anxiety-like behavior.

## 4. Discussion

Prolonged high-sugar intake is well-documented to adversely affect health [2,48,49,50]. Previous studies have shown that rodents exposed to high-sugar diets for 8 to 12 weeks can develop anxiety-like behaviors [51,52,53]. We now demonstrate that a significantly shorter dietary intervention, consisting of only 2 weeks of access to sugary beverages, is sufficient to induce these behaviors. This finding not only confirms the anxiogenic potential of high-sugar diets but also establishes a brief-exposure model for studying diet-induced anxiety. These findings suggest that even a relatively brief period of sugar access is sufficient to elicit anxiety-like behavior in mice, supporting the need for future studies to explore whether analogous effects may occur in humans.

Previous research indicates that sugar-preference behavior stems not only from the conscious perception of sweetness, but also from visceral sugar sensing process, via a sugar-specific pathway distinct from general sweet taste perception [30,31,32,54]. To determine whether two weeks of sugar exposure-induced anxiety-like behavior is driven by caloric content, sweet taste, or other properties, we tested several substances in our anxiety model, including sugars (sucrose, glucose, and the glucose analog MDG) and the artificial sweetener Ace-K. We found that Ace-K did not induce anxiety-like behavior, whereas all tested sugars did. These results indicate that the anxiogenic effect is independent of sweet taste and is specifically triggered by sugar molecules. Notably, previous work has shown that neurons in the NTS, which are selectively activated by glucose but not by Ace-K, mediate sugar-preference behavior via a gut–brain axis [32]. Our c-Fos data align with these findings and further suggest that specific activation of the gut–brain axis may contribute to sugar-induced anxiety-like responses. Nevertheless, the proposed pathway linking peripheral sugar sensing to NTS activation, as well as its underlying mechanisms, requires further experimental validation.

Recent evidence started to elucidate the molecular machinery underlying gut-to-brain sugar sensing. A key study demonstrated that intestinal glucose activates vagal neurons via a pathway independent of canonical sweet taste receptors (T1R2/T1R3), and that the non-metabolizable glucose analog MDG also robustly activates NTS neurons, suggesting that the sensor recognizes the glucose molecule itself rather than downstream metabolic signals [32]. Consistent with these findings, a recent study showed that intestinal glucose activates the vagus nerve via the SGLT1 in a gut–brain neural circuit regulating sucrose preference [55]. In addition, glucose is known to directly modulate gastrointestinal vagal afferent neurons through ATP-sensitive potassium (KATP) channel closure [56], providing a complementary mechanism by which luminal glucose could be transduced into neural activity. Notably, KATP channels are also expressed in NTS neurons themselves, where they directly couple local glucose levels to neuronal excitability [57,58,59]. This raises the possibility that, in addition to peripheral vagal signaling, central glucose sensing within the NTS may contribute to the behavioral effects observed in the present study. It is worth noting, however, that MDG cannot enter glycolysis and therefore would not be expected to elevate intracellular ATP or close KATP channels. The fact that MDG nonetheless activated NTS neurons and induced anxiety-like behavior in the present study suggests that metabolic processing of glucose is not required for NTS activation and is more consistent with a transporter-based sensing mechanism via SGLT1 than with KATP-dependent metabolic sensing. Nevertheless, KATP channels may still contribute to the sustained or amplified neural response to metabolizable glucose under chronic exposure conditions. Future studies employing genetic ablation or pharmacological blockade of SGLT1 and KATP channels in vagal sensory neurons would help delineate their respective contributions to sugar-induced anxiety-like behavior.

Glutamatergic and GABAergic neurons represent the majority of neuronal subtypes in the NTS [60,61,62]. In this study, we observed that exposure specifically increased c-fos expression in glutamatergic, but not GABAergic, neurons. Using patch-clamp recordings, we further found that this exposure also enhanced the intrinsic excitability of glutamatergic neurons, confirming their selective activation. In addition, we observed that two-week sugar exposure increased the amplitude of sEPSCs in glutamatergic neurons. This suggests that postsynaptic transmission strength in the NTS is enhanced, indicating the occurrence of long-term plasticity at glutamatergic synapses, likely involving changes in postsynaptic receptors [63,64]. This finding aligns with previous reports showing that various pathophysiological and environmental stimulus can potentiate glutamatergic synapses in the NTS [65,66,67,68,69,70]. The precise molecular alterations underlying this plasticity will be investigated in future studies.

We found that chemogenetic activation of glutamatergic neurons in the NTS is sufficient to induce anxiety-like behavior, while their inhibition attenuates sugar-induced anxiety-like responses. This finding is consistent with prior reports that reducing glutamatergic neuronal activity in the NTS alleviates anxiety-like behavior in models of restraint stress [71] and hypobaric hypoxia [72], further supporting the notion of the NTS as a key hub in anxiety regulation [73,74]. While chemogenetic approaches enable valuable cell-type-specific manipulation, several potential confounds should be considered. DREADDs may exhibit cell-type-specific constitutive activity, altering membrane potential and ion channel function even in the absence of agonist [75,76], though this has so far been observed only in non-neuronal cells [77,78]. Moreover, systemic CNO is metabolized into clozapine, which is more blood–brain barrier permeable and has activity at endogenous receptors (including dopamine and serotonin receptors), potentially leading to off-target effects that could confound DREADD-related results [79,80,81]. In the present study, the use of identical CNO doses and DREADD-negative controls helped minimize false-positive interpretations [82], although future work using highly selective agonists such as deschloroclozapine (DCZ) or optogenetics will further strengthen these findings [83,84]. Glutamatergic neurons in the NTS are considered the primary output pathway, and they frequently co-release noradrenaline [34,85,86]. They project extensively to key anxiety-related brain regions, including the locus coeruleus [86,87] and bed nucleus of the stria terminalis [88,89]. Therefore, identifying the specific downstream circuit by which NTS glutamatergic neurons drive sugar-induced anxiety remains a critical direction for future research. Based on converging evidence from literature, several downstream pathways may serve as key mediators. Among the candidates is the ventral bed nucleus of the stria terminalis (vBNST), which receives dense noradrenergic input from NTS A2 neurons and represents a well-characterized node in sustained anxiety circuitry. Evidence hinting at a causal relationship comes from optogenetic studies, where inhibition of NTS→vBNST projections was found to alleviate anxiety-like behaviors [88]. Consistent with this, a projection from prolactin-releasing peptide-positive noradrenergic neurons in the NTS to the vlBNST has been shown to modulate conditioned avoidance, a process highly relevant to interoceptive anxiety [73]; additionally, nitric oxide signaling within the NTS may regulate norepinephrine release in the BNST during stress [89]. Notably, the BNST is preferentially associated with sustained, generalized anxiety rather than acute fear, which may make it particularly relevant to the relatively prolonged anxiety phenotype induced by sugar consumption. Additionally, the locus coeruleus (LC), the major source of forebrain norepinephrine, represents another putative pathway. Optogenetic evidence suggests that activation of visceral afferents may increase anxiety via an NTS→LC circuit, with norepinephrine-expressing LC neurons significantly activated by this input [87], and coordinated NTS-LC activation has been observed in mouse models of anxiety [71]. Given its broad projections to cortical and limbic regions and classical association with arousal and vigilance, the LC may contribute to the hypervigilant component of sugar-induced anxiety. While an NTS→central amygdala (CeA) glutamatergic projection has been identified [85], this pathway is primarily characterized in depression-like behaviors, and its specific role in anxiety remains less clear. Importantly, the CeA is classically linked to phasic, cue-dependent fear, in contrast to the BNST’s preferential involvement in sustained anxiety, a distinction that may be functionally relevant to the metabolic and interoceptive nature of sugar-induced anxiety. Future studies utilizing cell-type-specific circuit manipulations will be needed to clarify the relative contributions of these various pathways and determine whether they act in parallel or in series.

The results of this paper are inspirational and show great promise, but we acknowledge some potential shortcomings. First, the precise molecular mechanisms by which sugar intake enhance the excitability and synaptic plasticity of NTS glutamatergic neurons remain to be fully elucidated. Given that research in metabolic diseases has shown that a 12-week high-fat diet induces stable insulin resistance in C57BL/6J mice [90], the neuronal changes observed after our 2-week intervention may represent early neural adaptations preceding systemic metabolic dysfunction. Such adaptations could involve alterations in insulin signaling or mitochondrial function, as implicated in the development of insulin resistance and associated behavioral changes [90]. This potential link warrants further investigation. Second, the generalizability of our findings requires verification. The C57BL/6J strain used here exhibits a pronounced susceptibility to diet-induced metabolic disturbances compared to other strains (e.g., 129/Sv) [91]; thus, examining whether the observed phenomena persist across different genetic backgrounds is crucial. Third, while our data point to a sugar-specific effect distinct from sweet taste, we cannot definitively exclude whether the anxiogenic effect derives from caloric sugar *per se* or from energy intake in general. Whether other caloric -ose molecules (fructose, galactose, etc.) or low-insulinemic sugar substitutes (e.g., sucralose, tagatose, or sugar alcohols) elicit similar neural and behavioral responses also remain untested. Future studies should employ isocaloric non-sugar controls (e.g., fat emulsion) to distinguish between these possibilities, while systematically comparing various -ose molecules alongside low-insulinemic substitute compounds could help identify the exact molecular determinants necessary for triggering the pathway. Fourth, despite the robust behavioral and neural effects observed, a limitation of our current experimental design warrants consideration. Specifically, mice in the sugar-exposed group exhibited a 22% increase in total fluid intake relative to water controls. Because the NTS is a primary relay center for interoceptive signals, receiving dense vagal afferent inputs that are highly sensitive to gastric distension and fluid volume [92,93], it is plausible that the increased fluid volume and subsequent mechanical stretch of the stomach acted as secondary covariates contributing to the observed NTS glutamatergic activation. This interpretation is further complicated by evidence that anxiety and stress can themselves promote excessive fluid consumption [94,95], creating a bidirectional confound. However, it is important to contextualize this within normal physiological parameters. Extensive literature demonstrates that moderate, physiological gastric distension primarily engages vagal mechanoreceptors to signal satiety and meal termination, rather than driving aversive or anxiety-like states [96]. Because the increased fluid consumption in our paradigm occurred ad libitum over a 24 h period, the acute mechanical stretch likely remained well within the physiological range. Noxious or extreme gastric distension is typically required to recruit anxiogenic circuits [97]. Future investigations utilizing volume-matched fluid paradigms or targeted intragastric infusions will be necessary to definitively disentangle the mechanical effects of gastric distension from the chemical properties of sugar consumption. Fifth, a limitation of the current study is the absence of peripheral metabolic phenotyping, such as the measurement of body weight trajectories, fasting blood glucose, and plasma insulin levels. However, it is important to note that our paradigm utilized a relatively short-term sugar exposure of two weeks. Previous studies employing similar short-term paradigms have demonstrated that 2 weeks of sugar exposure does not typically induce significant alterations in gross body weight or baseline blood glucose [98,99], yet it is sufficient to drive rapid neurochemical adaptations and behavioral changes [100]. Furthermore, our data revealed that mice consuming the non-caloric sweetener Ace-K did not exhibit anxiety-like behaviors. This critical finding indicates that the anxiogenic effects observed in the sugar-exposed group are not merely a consequence of sweet taste perception but are specifically driven by the post-ingestive chemical properties of sugar. Nonetheless, future studies incorporating comprehensive metabolic profiling over extended timeframes will be valuable to fully disentangle the direct neurobiological effects of sugar from secondary metabolic shifts. Finally, future work would benefit from incorporating a broader panel of physiological metrics, such as HbA1c, glucose tolerance, plasma insulin, and organ-specific health markers, alongside more comprehensive behavioral analyses (e.g., sleep patterns, energy metabolism). Correlating these parameters with neural changes would strengthen the connection between peripheral metabolic state and central emotional circuits.

## 5. Conclusions

Collectively, these results suggest that a sugar-specific pathway may recruit NTS glutamatergic neurons, which appear to be necessary for the expression of sugar-induced anxiety-like behavior in mice. Our study thus provides a simplified model for high-sugar diet-induced anxiety and offers a novel perspective on the integration of peripheral metabolic states and central emotional processing via a bottom-up neural axis.

## Data Availability

The raw data supporting the conclusions of this article will be made available by the authors upon reasonable request without undue reservation.

## Supplementary Materials

The following supporting information can be downloaded at: https://www.mdpi.com/article/10.3390/biology15080646/s1, Figure S1: Cumulative drinking volume of water and sucrose solution over two weeks. The line graph shows the cumulative intake of water and a sucrose solution over a 2-week period. Data are presented as mean values of each cage for each group (*n* = 10–11 mice from 3 cages per group). Figure S2: Chemogenetic silencing and activation of neuronal activity in NTS glutamatergic neurons in acute brain slices. (A) Left: Viral strategy schematic showing stereotaxic injection of AAV2/9-CaMKIIα-hM4Di-eGFP (inhibitory DREADD) into the NTS to target CamkIIα^+^ glutamatergic neurons. Right: Representative current-clamp voltage trace demonstrating robust, complete inhibition of action potential firing in an hM4Di-expressing NTS neuron following bath application of CNO (5 μM, red bar). (B) Summary data of firing rates for neurons significantly inhibited by CNO (Paired Student’s *t*-test, *p* = 0.001, *n* = 6 neurons from 3 mice). (C) Left: Viral strategy schematic showing stereotaxic injection of AAV2/9-CaMKIIα-hM3Dq mCherry (excitatory DREADD) into the NTS to target CamkIIα^+^ glutamatergic neurons. Right: Representative current-clamp voltage trace demonstrating robust activation of action potential firing in an hM3Dq-expressing NTS neuron following bath application of CNO (5 μM, red bar) (D) Summary data of firing rates for neurons significantly excited by CNO (Paired Student’s *t*-test, *p* = 0.018, *n* = 5 neurons from 2 mice). Data are presented as mean ± SEM; * *p* < 0.05, ** *p* < 0.01.

## Abbreviations

The following abbreviations are used in this manuscript:

NTS	Nucleus of the Solitary Tract
MDG	Methyl-alpha-D-glucopyranoside
Ace-K	Acesulfame Potassium
OFT	Open Field Test
EPM	Elevated Plus Maze
PFA	Paraformaldehyde
PBS	Phosphate-Buffered Saline
NGS	Normal Goat Serum
DAPI	4′,6-Diamidino-2-Phenylindole
ACSF	Artificial Cerebrospinal Fluid
IR-DIC	Infrared Differential Interference Contrast
sEPSCs	Spontaneous Excitatory Postsynaptic Currents
AP	Action Potential
CNO	Clozapine-N-oxide
DREADD	Designer Receptors Exclusively Activated by Designer Drugs
