# Supplementary material for: High-Sugar Consumption Induces Anxiety-Like Behavior via Activating the Glutamatergic Neurons in the Nucleus of the Solitary Tract in Mice
Source: Biology (Basel). 2026 Apr 19;15(8):646. doi: 10.3390/biology15080646 (PMC13113027; doi:10.3390/biology15080646)
Supplement: Supplementary file 1 [file biology-15-00646-s001.zip › biology-4247902-supplementary.pdf]

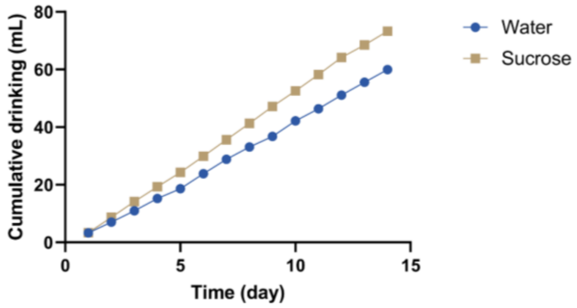

Supplemental Figure S1. Cumulative drinking volume of water and sucrose solution over two weeks.

The line graph shows the cumulative intake of water and a sucrose solution over a 2-week period. Data are presented as mean values of each cage for each group (n = 10–11 mice from 3 cages per group).

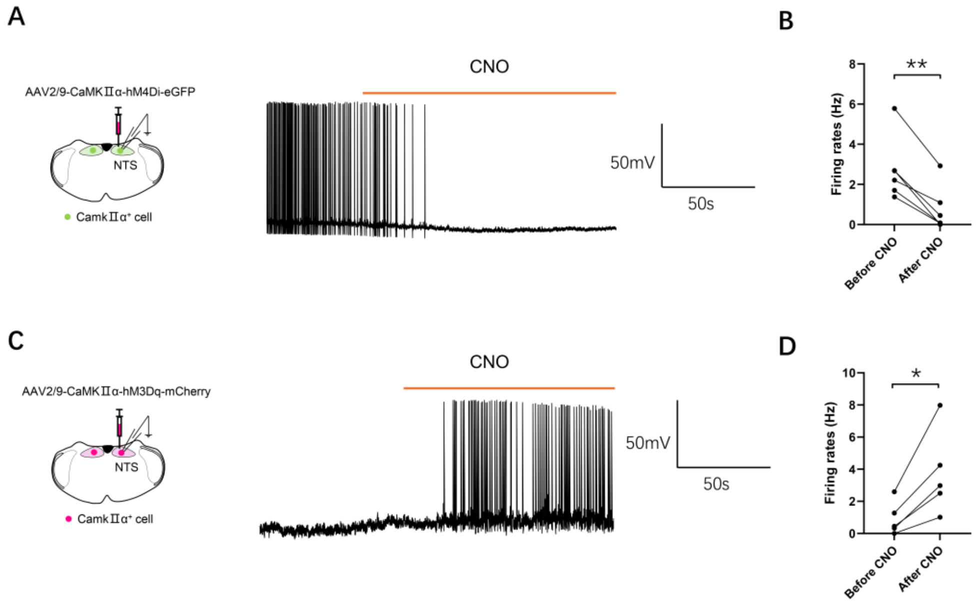

Supplemental Figure S2. Chemogenetic silencing and activation of neuronal activity in NTS glutamatergic neurons in acute brain slices.

(A) Left: Viral strategy schematic showing stereotaxic injection of AAV2/9-CaMKIIα-hM4Di-eGFP (inhibitory DREADD) into the NTS to target CamkIIα$^+$ glutamatergic neurons. Right: Representative current-clamp voltage trace demonstrating robust, complete inhibition of action potential firing in an hM4Di-expressing NTS neuron following bath application of CNO (5 μM, red bar).

(B) Summary data of firing rates for neurons significantly inhibited by CNO (Paired Student's *t*-test, $p = 0.001$, $n = 6$ neurons from 3 mice).

(C) Left: Viral strategy schematic showing stereotaxic injection of AAV2/9-CaMKIIα-hM3Dq-mCherry (excitatory DREADD) into the NTS to target CamkIIα$^+$ glutamatergic neurons. Right: Representative current-clamp voltage trace demonstrating robust activation of action potential firing in an hM3Dq-expressing NTS neuron following bath application of CNO (5 μM, red bar)

(D) Summary data of firing rates for neurons significantly excited by CNO (Paired Student's *t*-test, $p = 0.018$, $n = 5$ neurons from 2 mice).

Data are presented as mean ± SEM; $*p < 0.05$, $**p < 0.01$.
